# The neuroprotective mechanism of sevoflurane in rats with traumatic brain injury via FGF2

**DOI:** 10.1186/s12974-021-02348-z

**Published:** 2022-02-17

**Authors:** Zhongyu Wang, Zhaoyang Wang, Anqi Wang, Juan Li, Junmin Wang, Jingjing Yuan, Xin Wei, Fei Xing, Wei Zhang, Na Xing

**Affiliations:** 1 Department of Anesthesiology and Perioperative Medicine, the First Affiliated Hospital of Zhengzhou University, No. 1, Jianshe East Road, Zhengzhou, 450052 Henan Province People’s Republic of China; 2 Henan Province International Joint Laboratory of Pain, Cognition and Emotion, the First Affiliated Hospital of Zhengzhou University, No. 1, Jianshe East Road, Zhengzhou, 450052 Henan Province People’s Republic of China; 3 Department of Human Anatomy, Basic Medical College of Zhengzhou University, Zhengzhou, 450001 Henan Province People’s Republic of China

**Keywords:** Traumatic brain injury, Sevoflurane, FGF2, EZH2, HES1, Neuroprotection, Neuronal apoptosis, Neurological deficits

## Abstract

**Background:**

Traumatic brain injury (TBI) is a kind of acquired brain injury, which is caused by external mechanical forces. Moreover, the neuroprotective role of sevoflurane (Sevo) has been identified in TBI. Therefore, this research was conducted to figure out the mechanism of Sevo in TBI via FGF2.

**Methods:**

The key factors of neuroprotective effects of Sevo in TBI rats were predicted by bioinformatics analysis. A TBI model was induced on rats that then inhaled Sevo for 1 h and grouped via lentivirus injection. Modified Neurological Severity Score was adopted to evaluate neuronal damage in rats, followed by motor function and brain water content measurement. FGF2, EZH2, and HES1 expression in brain tissues was evaluated by immunofluorescence staining, and expression of related genes and autophagy factors by RT-qPCR and Western blot analysis. Methylation-specific PCR was performed to assess HES1 promoter methylation level, and ChIP assay to detect the enrichment of EZH2 in the HES1 promoter. Neuronal damage was assessed by cell immunofluorescence staining, and neuronal apoptosis by Nissl staining, TUNEL staining, and flow cytometry.

**Results:**

Sevo diminished brain edema, improved neurological scores, and decreased neuronal apoptosis and autophagy in TBI rats. Sevo preconditioning could upregulate FGF2 that elevated EZH2 expression, and EZH2 bound to the HES1 promoter to downregulate HES1 in TBI rats. Also, FGF2 or EZH2 overexpression or HES silencing decreased brain edema, neurological deficits, and neuronal autophagy and apoptosis in Sevo-treated TBI rats.

**Conclusions:**

Our results provided a novel insight to the neuroprotective mechanism of Sevo in TBI rats by downregulating HES1 via FGF2/EZH2 axis activation.

**Supplementary Information:**

The online version contains supplementary material available at 10.1186/s12974-021-02348-z.

## Background

Traumatic brain injury (TBI) is a kind of acquired brain injury, which is caused by external mechanical force, including bump, blow, or jolt to the head, or a penetrating head injury, and can lead to temporary or permanent injury [1]. More than 50 million people suffer from TBI every year on a global scale, half of which may suffer from one or more kinds of TBI in their lifetime [2]. Unfortunately, in the case of TBI, even transient hypoperfusion and hypoxemia can lead to secondary injury and further worse short-term and long-term outcomes [3]. Because of its high morbidity and long-term sequelae, TBI results in great elevation of health care expenditure costs every year [4]. Moreover, TBI has been documented to cause neurological deficits, behavioral alterations, and cognitive decline and impose a dramatic impact on patients [5]. However, despite advances in developing therapeutic strategies on TBI recovery, effective treatments for TBI recovery was lack currently [6]. Hence, there is ongoing need for exploration of molecular mechanism underlying TBI to figure out more effective treatment of TBI.

As a halogenated inhalational anesthetic, sevoflurane (Sevo) is approved by FDA for induction and maintenance of general anesthesia in adult and pediatric inpatients and outpatients undergoing surgery, which offers autonomic blockade, hypnosis, analgesia, amnesia, and akinesia during surgical and procedural interventions [7]. Furthermore, Sevo postconditioning has been identified to alleviate TBI by reducing neuronal apoptosis and promoting autophagy [8]. Interestingly, it was predicted in our study by microarray analysis that fibroblast growth factor 2 (FGF2) may be a key factor in the neuroprotective effect of Sevo on TBI. FGF2 (also named as basic fibroblast growth factor) is a 3-exon gene on human chromosome 4q26-27, which possesses low (18-kDa) and high (22-, 22.5-, 24-, and 34-kDa) molecular weight isoforms and are translated from a single transcript by starting from alternative, in-frame start codons [9]. Importantly, a prior research exhibited that FGF2 could protect against blood–brain barrier damage in mice with TBI [10]. Intriguingly, another work elucidated that FGF2 can increase enhancer of zeste homolog 2 (EZH2) expression by activating KDM2B in bladder cancer cells [11]. More importantly, Sevo-upregulated EZH2 was capable of alleviating hypoxic–ischemic cerebral injury in neonatal rats [12]. In addition, it was documented that EZH2 was involved in the transient repression of hairy and enhancer of split 1 (HES1) in erythroid cells [13]. Notably, knockdown of HES1 was able to augment the spatial learning and memory capacity of adult mice with TBI [14].

In this context, we speculated that the FGF2/EZH2/HES1 axis might be correlated with the neuroprotective effect of Sevo on TBI, and conducted a series of animal and cell experiments to verify this speculation.

## Methods

### Ethics statement

Animal experiments were approved by the Ethics Committee of the First Affiliated Hospital of Zhengzhou University and conducted strictly in line with the Guide for the Care and Use of Laboratory Animals published by the US National Institutes of Health. All efforts were made to minimize the number and suffering of the included animals.

### Microarray analysis

Gene expression dataset related to Sevo-treated rat brain tissues, GSE141242, was retrieved from the Gene Expression Omnibus (GEO) database (the platform annotation file was GPL22388 [RTA-1_0] Affymetrix Rat Transcriptome Array 1.0 [transcript (gene) CSV version]), including three control samples (sham) and three treated samples (TBI models). Differential analysis was conducted using “limma” package of R language to screen differentially expressed genes (DEGs) with |log2FC|> 0.6 and *p* < 0.05 as screening criterion. R language “pheatmap” package was adopted to draw a heat map of DEG expression. In the GeneCards database (score ≥ 18), “TBI” was employed as a keyword to search for TBI-related genes, which were then intersected with DEGs in Sevo-treated rat brain tissues using jvenn to figure out the central factors involved in the neuroprotective effects of Sevo on TBI in rats.

### Rat TBI model construction and lentivirus treatment

Healthy adult male Sprague Dawley rats (weighing 200–220 g; aged 3–4 months; the Experimental Animal Center of Zhengzhou University) were housed at 20–24 °C with 40–60% of humidity, 12 h/day light, and free access with water and food.

The rats were randomly assigned into 13 groups (Additional file 1: Table 1) in accordance with the random number table, with 12 rats in each group. Postoperatively, they were subdivided into 1-, 3-, 7-, and 14-day groups, with three rats in each group. A modified Feeney’s free-falling epidural percussion method was utilized to establish TBI model to induce brain injury in rats. Following anesthesia of rats by intraperitoneal injection of 3% pentobarbital sodium (50 mg/kg), the scalp was cut open at 2 mm behind the right coronal suture and 2 mm in the midline. A 5-mm hole was drilled in the skull, but the dura was intact. A 30-g hammer was thrown down from a height of 20 cm to cause craniocerebral injury (impact force = 600 g/cm). The bone holes were sealed with wax, and the scalp was sutured. The sham-operated rats underwent this surgical procedure without hammering. Each rat was placed in a 42 × 26 × 26 cm closed anesthesia box. Anesthesia holes at 1.5 cm diameter were on both sides of the box for gas input and removal.

At 30 min after model construction, pure oxygen was delivered into sham-operated and TBI rats, and after 60 min, it was removed. TBI rats treated with Sevo were given with 2.4% Sevo-containing oxygen for 60 min. After additional 15-min oxygen inhalation, the rats were removed from the box. The gas analyzer was used to monitor the concentration of Sevo, oxygen and carbon dioxide. Following 0.5-h Sevo exposure, the right ventricle of rats was injected with 600 nmol 3-methyladenine (3-MA, diluted in 0.9% normal saline to a final volume of 5 μL), an inhibitor of cell autophagy that specifically blocks autophagosome formation and is utilized to consolidate the role of the autophagic pathway in the adaptive neuroprotection following Sevo treatment. The remaining rats were injected with 0.9% normal saline as a control. The rats were euthanized at the end of the experiment, and the cortical tissue was collected and sectioned for subsequent experiments.

Two days before TBI model construction, the rats were immobilized under a stereotaxic frame (RWD, Shenzhen, China) and anesthetized with sodium pentobarbital (3%). The left ventricle (anterior–posterior-1.1 mm, medial–lateral-1.5 mm, dorsal–ventral-4.0 mm from the bregma) of rats was injected with 4 μL of each lentivirus at a titer of 2 × 10^8^ ifu/mL using a stereotaxic instrument (at a rate of 1 μL/min, with 5–10 min of retaining needle). After 2 days, model construction was performed [15].

### Isolation and incubation of hippocampal neurons

Healthy female Sprague–Dawley rats on day 17 of pregnancy were routinely anesthetized, disinfected and dissected, after which the fetal rats were taken out and placed in Dulbecco’s modified Eagle’s medium (DMEM). Under an anatomic microscope, dissecting tweezers were used to remove the hippocampal tissue, with the meninges and superficial vessels completely discarded to obtain the hippocampal tissues. The hippocampal tissues were cut into pieces with ophthalmic scissors, detached with 0.125% trypsin, and reacted in a 37 °C water bath for 30 min, during which shaking 2–3 times was performed. When the digestive juice was turbid and did not contain tissue mass, the digestion was terminated by DMEM containing 10% fetal bovine serum. The tissue pieces were then centrifuged at 1500 r/min for 5 min, and the supernatant was discarded, followed by another centrifugation by addition of stop solution. Following supernatant removal, the pellet was added with stop solution again, and gently dispersed with a micropipette with very small inner diameter until a uniform cell suspension formed. Cell suspension was filtered with a 200 mesh filter to remove undigested tissue fragments, and the filtered single cell suspension was collected in a beaker. Next, the suspension was stained with trypan blue, counted on a hemocytometer, plated in a culture plate at a density of 1 × 10^5^ cells/mL, and cultured in a 5% CO_2_ incubator at 37 °C. After 8 h, the culture medium was renewed and cells continued to culture.

### Culture of 293T cells

293T cells (the Cell Bank of the Type Culture Collection Committee of the Chinese Academy of Sciences) were seeded in a 25-cm^2^ culture flask at a density of 1 × 10^5^ cells/mL, and cultured in a 5% CO_2_ incubator at 37 °C. When reaching 60–70% confluence, the cells were passaged for subsequent experiments.

### *Transduction of rat hippocampal neurons and establishment of *in vitro* TBI models*

Cells in good conditions were screened under a microscope and transduced with lentivirus using Lipofectamine 2000 reagent for 72–96 h. Control cells were treated with 21% O_2_ and 5% CO_2_ for 3 h and Sevo cells were treated with 4.1% Sevo, 21% O_2_ and 5% CO_2_ for 3 h. The TBI cell model was established as previously described [16]. Briefly, a yellow pipette tip (1.5 mm in diameter) and a white pipette tip (1 mm in diameter) were used to mechanically cut the cultured rat hippocampal neurons to establish a TBI cell model injured by mechanical force.

### Neurological function evaluation

The Modified Neurological Severity Score (mNSS) was adopted for evaluation of motion, sensation, reflex, muscle mass, abnormal behavior, vision, touch, and balance of rats, which was graded on a scale of 0–18. A normal state was illustrated by 0 score, and severe neurological deficits was indicated by 18 scores. The higher score indicated the greater neurological damage. Neurological function evaluation was conducted on day 1, 3, 7, and 14 following TBI modeling [8, 17]. The wire grip test was performed to determine the motor function of rats [18]. Each rat was placed on a horizontal wire (80 cm in length and 7 mm in diameter) 45 cm away from the ground, and was allowed to crawl freely on the wire within 60 s. Bermpohl’s method was applied to score motor function, ranging from 0 to 5 points, a total of six grades and evaluation was conducted on the 1, 3, 7 and 14 days after TBI. The lower score reflected the more severe impairment of motor function.

### Measurement of brain water content

Following neurological function assessment, three rats in each group were euthanized at 1, 3, 7, and 14 days, respectively, under deep anesthesia, followed by removal of the brain. A precision electronic scale was used to weigh the brain tissue as “wet weight” and the weighed brain tissue was then put in a suitable container and in a 120 °C oven for about 48 h. During this process, the weight was measured several times until no changes occurred, which was the “dry weight”. The brain water content was calculated using the following formula: brain water content = (wet weight − dry weight) × 100% [8, 17].

### Nissl staining

Cortical tissues surrounding the lesion areas were harvested, followed by formaldehyde fixing and preparation of 4-μm paraffin-embedded sections. The sections were dewaxed with xylene, rehydrated in a graded series of alcohol, and cultured with Nissl staining solution for 5 min. The damaged neurons shrank or contained vacuoles, and the normal neurons had a relatively large and full soma, with round and large nuclei. Neurons were counted under a microscope in five randomly selected visual fields.

### Terminal deoxynucleotidyl transferase-mediated dUTP-biotin nick end labeling (TUNEL) staining

Cortical tissues were collected, fixed with 4% paraformaldehyde for 24 h and rinsed with running water. Next, the tissues were dehydrated in ascending series of alcohol (70%, 80%, 90%, 95% and 100%) for 30 min, cleared with xylene, embedded in paraffin and cut into 4-μm-thick sections. The sections were stained with TUNEL cell apoptosis kit (C1086, Beyotime Biotechnology Co., Shanghai, China) and apoptotic cells were observed under an inverted fluorescence microscope (HB050; Zeiss, Hamburg, Germany) in six randomly selected visual fields from each section. The percentage of the number of apoptotic cells to the total number of cells was the apoptosis rate.

### Tissue immunofluorescence staining

Cortical tissues around the lesion area were fixed with formaldehyde and embedded in paraffin to prepare 3-μm-thick paraffin sections. The sections were deparaffinized by xylene, hydrated with gradient alcohol, and boiled in citric acid buffer for 5 min for antigen retrieval. The sections were then blocked with 5% goat serum and incubated overnight with primary antibodies against FGF2 and EZH2 (detailed information is shown in Additional file 1: Table 2). Following phosphate-buffered saline (PBS) washing, the sections were incubated with secondary antibody goat anti-rabbit IgG (1:200) conjugated by fluorescein isothiocyanate for 30 min. The nuclei were stained with 4′,6-diamidino-2-phenylindole (DAPI) and then observed under a fluorescence microscope [8, 12, 17].

### Flow cytometry

A total of 1 × 10^6^ cells were added with 80 μL RNase (0.03 g/L) and 150 μL propidium iodide (PI) (0.05 g/L, containing 0.03% Triton X-100), and reacted at 4 °C for 30 min in the dark. A flow cytometer was used to detect the percentage of cells in different cell cycles. CellQuest software was adopted to collect cells, at least 1 × 10^4^ cells, and ModFit software was used to analyze the results, which were expressed as percentage [19].

### Western blot analysis

The cortical tissues (30 mg) and cells surrounding the lesion area were lysed in lysis buffer, followed by quantification of the total protein concentration using a bicinchoninic acid kit. After the lysate was added to the reduced loading buffer, samples were prepared and boiled for 8 min. Next, 25 μg total protein was loaded per sample before protein separation by sodium dodecyl sulfate–polyacrylamide gel electrophoresis. The protein was electroblotted to a polyvinylidene fluoride membrane which was blocked in 5% skim milk at room temperature for 2 h. The membrane was probed with corresponding primary antibodies (Additional file 1: Table 2) at 4 °C overnight, and re-probed with horseradish peroxidase-conjugated secondary goat anti-rabbit antibody (1:5000; ab6721, Abcam) at room temperature on the next day. The protein bands were visualized by enhanced chemiluminescence. Image J software was applied for gray-scale quantification of protein bands with glyceraldehyde-3-phosphate dehydrogenase (GAPDH) as a normalizer.

### Cell immunofluorescence analysis

The sterile glass slide was placed into a 24-well plate to make cover glasses, which were coated with polylysine. The cultured neurons were digested into a single cell suspension, seeded on the slide and cultured with cell culture medium in a cell incubator with constant temperature.

After 48 h, the cells were observed under a microscope. When reaching about 80% confluence, the cells were fixed with 3% paraformaldehyde in ice at room temperature for 15 min, blocked with 3% bovine serum albumin (BSA), and incubated in PBS for 1 h. Thereafter, the cells were probed with primary antibody to MAP2 (1:50, ab183830, Abcam) at 4 °C overnight. After washing with PBS, the cells were re-probed with fluorescein isothiocyanate-labeled secondary antibody (goat anti-rabbit, 1:100, A-10684, Invitrogen Inc., Carlsbad, CA, USA) in PBS in the dark for 1 h at room temperature. Next, the cover glass was added with a mounting tablet containing DAPI (1 μg/mL), then removed from the 24-well plate, buckled upside down on the cover glass and mounted. A laser confocal microscope was used to analyze the results.

Fluorescence staining of neuronal axons was conducted as previously described [20]. Hippocampal neurons at the logarithmic growth phase were selected for follow-up experiments. The neurons were cultured for 48 h, and fixed for immunofluorescence labeling. Axons (by Tau-1) and dendrites (by MAP2) were labeled, respectively. Immunofluorescence double-labeling: hippocampal neurons were cultured for 48 h, added with 4% paraformaldehyde and fixed at room temperature for 15–20 min, rinsed with PBS-0.1% Triton, then treated with PBS-0.5% Triton for 5–10 min, rinsed with PBS-0.1% Triton, blocked with 5% BSA for 1 h, incubated with mouse anti-MAP2 (1:200) or mouse anti tau-1 (1:200), respectively, overnight at 4 °C. After rinsing, fluorescent-labeled antibody II was added to the neurons and incubated at room temperature for 1 h, and then observed under confocal microscope. Image Proplus software was used to count the protrusion length. Firstly, the measured length unit was standardized according to the ruler (20 μm) provided in the picture, and then the length of each segment of neuronal axon was measured with the standardized measuring ruler. After the measured data were exported, the total axon length was calculated in excel table and the data were recorded. The axon length and number of 50 neurons in each group were counted, respectively, and then the data were statistically analyzed. Student’s *t* test was used for comparison between the two groups, *p* < 0.05 represented significant difference. The average axon length of each group was calculated and a columnar analysis was conducted.

### Reverse transcription quantitative polymerase chain reaction (RT-qPCR)

Total RNA was extracted from tissues or cells using TRIzol reagent (15596026, Invitrogen), followed by reverse transcription into cDNA following the manuals of a PrimeScript RT reagent Kit (RR047A, Takara, Tokyo, Japan). The synthesized cDNA was subjected to RT-qPCR using the Fast SYBR Green PCR kit (Applied Biosystems, Carlsbad, CA, USA) on an ABI PRISM 7300 RT-PCR system (Applied Biosystems). Three replicates were set up for each well. The gene relative expression was calculated using the 2^−ΔΔ*Ct*^ method and standardized by GAPDH. The primer sequences are depicted in Additional file 1: Table 3.

### Chromatin immunoprecipitation (ChIP) assay

National Center for Biotechnology Information (https://www.ncbi.nlm.nih.gov/) and JASPAR database (http://jaspar.genereg.net/) were adopted to predict binding sites of EZH2 in the HES1 promoter. ChIP assay was performed as per the kit instructions (Millipore, Billerica, MA, USA) as well as previously reported experimental methods. Cells were cross-linked in 1% formaldehyde for 10 min at 37 °C and then sonicated to obtain chromatin fragments averaging 400–800 bp. A small portion of the sample was applied as input, while the rest was added with anti-EZH2 antibody (ab191250, 1:1000, Abcam) and IgG (Santa Cruz Biotechnology Inc., Santa Cruz, CA, USA) as negative control (NC) for overnight incubation at 4 °C. The immunocomplexes were precipitated by supplementing protein A immunomagnetic beads the following day in the metal bath at 68 °C for 2 h for de-crosslinking. DNA was extracted using phenol chloroform isopropanol and quantitatively analyzed by RT-qPCR. One of the primers was a specific promoter for HES1 and the other was specific to NC (kidney specific promoter: Tamm–Horsfall). The primer sequences are displayed in Additional file 1: Table 4.

### Methylation-specific PCR (MSP)

DNA extraction: 3 days after modeling, the rats were euthanized to obtain brain tissues. DNA methylation treatment was performed using the EZ DNA Methylation-Gold™ Kit (Zymo Research Corp., USA) and the required DNA was then isolated.

PCR amplification: agarose gel electrophoresis was used to verify the extracted product and DNA Methylation-Gold™ Kit was applied to modify the product, followed by PCR amplification. The primers for HES1 gene methylation (Additional file 1: Table 5) and non-methylation (Additional file 1: Table 4) were synthesized by referring to relevant literature. Methylation level of CpG island in HES1 promoter region were determined by MSP. PCR was performed in a total volume of 25 µL encompassing 80 ng DNA template with the following reaction conditions: predenaturation at 95 °C for 5 min, 35 cycles of denaturation at 95 °C for 30 s, annealing at 55 °C for 30 s, and extension at 72 °C for 30 s, and extension at 72 °C extension for 10 min. Nuclease-free water served as a NC. Samples of PCR products (172 bp for methylated and 175 bp for unmethylated) were visualized on a 2% agarose gel containing 5 mg/mL ethidium bromide, and analyzed and photographed under ultraviolet irradiation using ChemiDoc™ MP imaging system (Bio-Rad Laboratories Inc., Hercules, CA, USA).

### Dual luciferase reporter assay

Constructs of the 3′untranslated region (UTR) dual luciferase reporter vectors of HES1 and mutant plasmids of mutations in the binding sites of HES1 to EZH2 were made as follows: PmirGLO-HES1-wild type (WT) and PmirGLO-HES1-mutant type (MUT), respectively. The reporter plasmids were, respectively, co-transfected with overexpression (oe) EZH2 plasmid and oe NC plasmids into 293T cells that were lysed after 48 h and centrifuged at 12,000×*g* for 1 min with the supernatant harvested. Luciferase activity was detected on a Dual-Luciferase® Reporter Assay System (E1910, Promega, Madison, WI, USA). The relative luciferase activity was calculated as the ratio of relative luciferase activity of firefly luciferase to that of renilla luciferase.

### Statistical analysis

Measurement data were summarized as mean ± standard deviation. Statistical analysis for all data in the current study was conducted using SPSS 21.0 software (IBM Corp., Armonk, NY, USA), using a value of *p* < 0.05 as an equivalent of statistical significance. Data among multiple groups were compared by one-way analysis of variance (ANOVA), and neurological scores and brain water content of rats at different time points were compared by two-way ANOVA, followed by Tukey’s post hoc test. Neurological function score and motor function score were grade data, and were assessed by nonparametric test.

## Results

### Sevo alleviated neuronal damage and apoptosis to reduce TBI-induced neurological deficits in rats

**Fig. 1:**
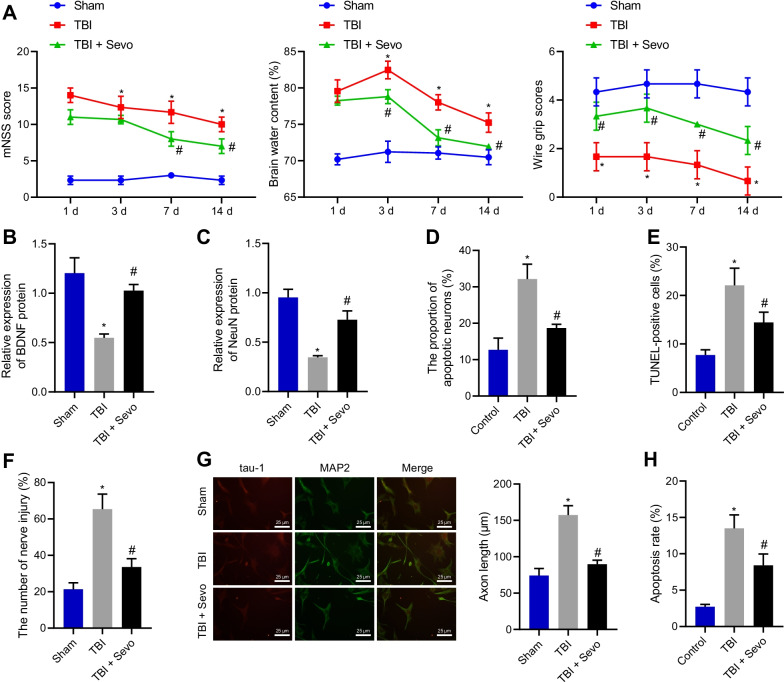
Sevo causes attenuation of neurological deficits and neuronal apoptosis in TBI rats. The sham-operated rats were utilized as controls, and TBI rats were treated or untreated with Sevo. **A** Neurological function evaluation by mNSS, brain water content measurement and motor function score in rats. **B** The protein expression of BDNF detected by Western blot analysis in the cortical tissue of rats. **C** Western blot analysis of the protein expression of NeuN in the cortical tissue of rats. **D** Nissl staining of the hippocampal neuronal damage in the cortical tissue of TBI rats. **E** TUNEL-positive cells in the cortical tissue of TBI rats. **F** Neuronal damage assessed by cell immunofluorescence assay (After primary hippocampal neurons were cultured for 48 h, Tau-1 was used to specifically identify axons, with red fluorescent markers in the figure; MAP2 was used to specifically identify dendrites, with green fluorescent markers, with a scale of 20 μm). **G** Axonal length measurement of hippocampal neurons. **H** The apoptosis of hippocampal neurons detected by flow cytometry. In panel **A**–**E** *n* = 12 for rats upon each treatment. *p < 0.05 vs. sham-operated rats or control cells; ^#^*p* < 0.05 vs. TBI rats or cells. Cell experiments were conducted three times independently

In order to study the effect of Sevo on TBI, a TBI model was induced in rats that were then treated with Sevo. As depicted in Fig. 1A, TBI rats had higher mNSS and brain water content yet lower motor function score than sham-operated rats. In contrast, Sevo treatment reduced mNSS and brain water content while increasing motor function score. As reflected by Western blot analysis, brain-derived neurotrophic factor (BDNF) and NeuN expression was lower in the cortical tissue of TBI rats than in sham-operated rats, which was reversed by Sevo treatment (Fig. 1B, C; Additional file 2: Fig. S1A, B). Nissl staining results showed that compared with sham-operated rats, the cortical tissue of TBI rats showed severe neuronal damage, which was alleviated by Sevo treatment (Fig. 1D). TUNEL staining data further displayed that in contrast to sham-operated rats, cell apoptosis was higher in the cortical tissue of TBI rats, while it was decreased upon the treatment of Sevo (Fig. 1E). In addition, MAP2 was utilized as a DNA damage marker, and neuronal damage was detected by cell immunofluorescence staining, with the results showing augmented neuronal damage in TBI rats while Sevo resulted in alleviation (Fig. 1F). Moreover, axonal length was found to be significantly longer in TBI rats than in sham-operated rats, and was shorter in Sevo-treated TBI rats than in TBI rats (Fig. 1G). Based on flow cytometric data, neuron apoptosis was elevated in TBI rats versus sham-operated rats, which was negated by Sevo treatment (Fig. 1H). Conclusively, Sevo reduced neuronal damage and apoptosis, improving neurological scores in TBI rats.

### Sevo promoted FGF2 expression and in turn attenuated neurological deficits and neuronal death caused by TBI in rats

**Fig. 2:**
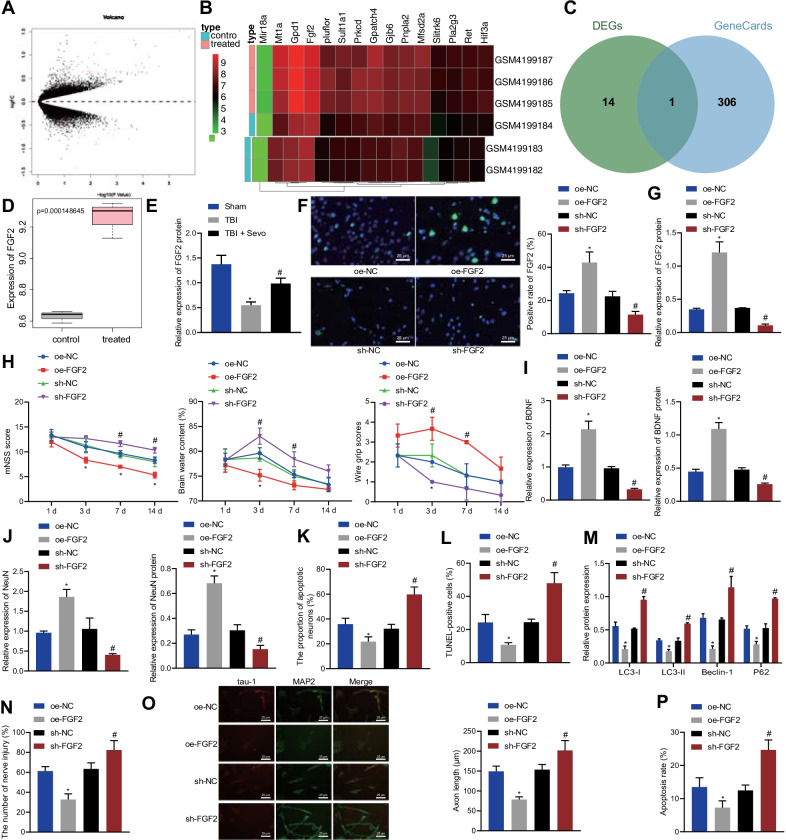
Sevo activates FGF2 to relieve neurological deficits and neuronal death in TBI rats. **A** Differential analysis of the GSE141242 dataset related to Sevo-treated rat brain tissues. **B** The expression heat map of the top 15 DEGs with the smallest *p*-value. **C** Intersection of the top 15 DEGs with the smallest *p*-value with TBI-related genes obtained through GeneCards database. **D** FGF2 expression in TBI rats after Sevo treatment analyzed by gene microarray data. **E** Western blot analysis of FGF2 expression in the cortical tissue of sham-operated, TBI, or Sevo-treated TBI rats. Sevo-treated TBI rats were treated with sh NC, sh FGF2, oe NC, or oe FGF2. **F** Immunofluorescence analysis of FGF2 expression in the cortical tissue of TBI rats (scale bar: 25 μm). **G** Western blot analysis of FGF2 expression in the cortical tissue of TBI rats. **H** Neurological function evaluation by mNSS, brain water content measurement and motor function score in TBI rats. **I** The expression of BDNF determined by Western blot analysis and RT-qPCR in the cortical tissue of TBI rats. **J** RT-qPCR detection and Western blot analysis of the expression of NeuN in the cortical tissue of TBI rats. **K** Nissl staining of the hippocampal neuronal damage in the cortical tissue of TBI rats. **L** TUNEL-positive cells in the cortical tissue of TBI rats. **M** Western blot analysis of protein expression of autophagy-related genes (LC3-I, LC3-II, Beclin-1, and P62) in the cortical tissue of TBI rats. TBI hippocampal neurons were treated with oe NC, sh NC, oe FGF2 or sh FGF2. **N** Neuronal damage assessed by cell immunofluorescence assay. **O** Axonal length measurement of hippocampal neurons (After primary hippocampal neurons were cultured for 48 h, Tau-1 was used to specifically identify axons, with red fluorescent markers in the figure; MAP2 was used to specifically identify dendrites, with green fluorescent markers, with a scale of 25 μm). **P** The apoptosis of hippocampal neurons measured by flow cytometry. In panel **E**–**M**, *n* = 12 for rats upon each treatment. *p < 0.05 vs. sham-operated rats, Sevo-treated TBI rats or hippocampal neurons treated with oe NC; ^#^*p* < 0.05 vs. TBI rats, Sevo-treated TBI rats or hippocampal neurons treated with sh NC. Cell experiments were conducted three times independently

To predict the key factors responsible for the neuroprotective effects of Sevo in TBI rats, we performed differential analysis of GSE141242 related to Sevo-treated rat brain tissues, which obtained 65 DEGs, including 41 high-expressed genes and 24 low-expressed genes (Fig. 2A). Then, we plotted the expression heat map of the top 15 DEGs with the smallest *p*-value (Fig. 2B). Totally 307 TBI-related genes were obtained through GeneCards database, which was intersected with the top 15 DEGs with the smallest *p*-value, finally obtaining FGF2 (Fig. 2C). Therefore, we presumed that FGF2 might be a key factor in the neuroprotective effect of Sevo on TBI rats.

Gene microarray data analysis showed that Sevo enhanced FGF2 expression in TBI rats (Fig. 2D). Western blot analysis results further presented a decline in the FGF2 protein expression in the cortical tissue of the TBI rats while Sevo treatment resulted in an increase in the FGF2 protein expression (Fig. 2E; Additional file 2: Fig. S1C). The results of immunofluorescence assay and Western blot analysis showed that the FGF2 expression was increased in the cortical tissue of the oe FGF2-treated rats, but a contrary result was noted in the absence of FGF2 (Fig. 2F, G; Additional file 2: Fig. S1D). As documented in Fig. 2H, overexpression of FGF2 diminished mNSS and brain water content while increasing motor function score in Sevo-treated TBI rats, which was opposite after silencing of FGF2. Moreover, the results of RT-qPCR and Western blot analysis indicated that FGF2 overexpression enhanced the expression of BDNF and NeuN in the cortical tissue of Sevo-treated TBI rats, but FGF2 silencing resulted in opposite results (Fig. 2I, J; Additional file 2: Fig. S1E, F). Additionally, Nissl staining results exhibited that FGF2 overexpression reduced the degree of neuronal damage in the cortical tissue of Sevo-treated TBI rats while FGF2 silencing enhanced the neuronal damage (Fig. 2K). TUNEL staining data presented a decline in the neuronal apoptosis in the cortical tissue of Sevo-treated TBI rats following FGF2 overexpression, which was opposite after silencing FGF2 (Fig. 2L). In addition, Western blot analysis showed that sh FGF2 treatment resulted in upregulation of autophagy-related genes (LC3-I, LC3-II, Beclin-1, and P62) but oe FGF2 treatment caused downregulation of these genes in Sevo-treated TBI rats (Fig. 2M).

Moreover, the results of cell immunofluorescence staining revealed that FGF2 overexpression reduced the hippocampal neuron damage, which was aggravated following FGF2 silencing (Fig. 2N). The results in Fig. 2O suggested that axonal length was shortened by overexpressing FGF2, but lengthened by silencing FGF2. Flow cytometric data presented with reduction of neuronal apoptosis after overexpressing FGF2, the effect of which reversed by silencing of FGF2 (Fig. 2P). Collectively, Sevo pretreatment can upregulate FGF2, thus decreasing neuronal autophagy and apoptosis, as well as attenuating neurological deficits in TBI rats.

### Sevo augmented EZH2 expression via FGF2 and then alleviated neurological deficits caused by TBI in rats

**Fig. 3:**
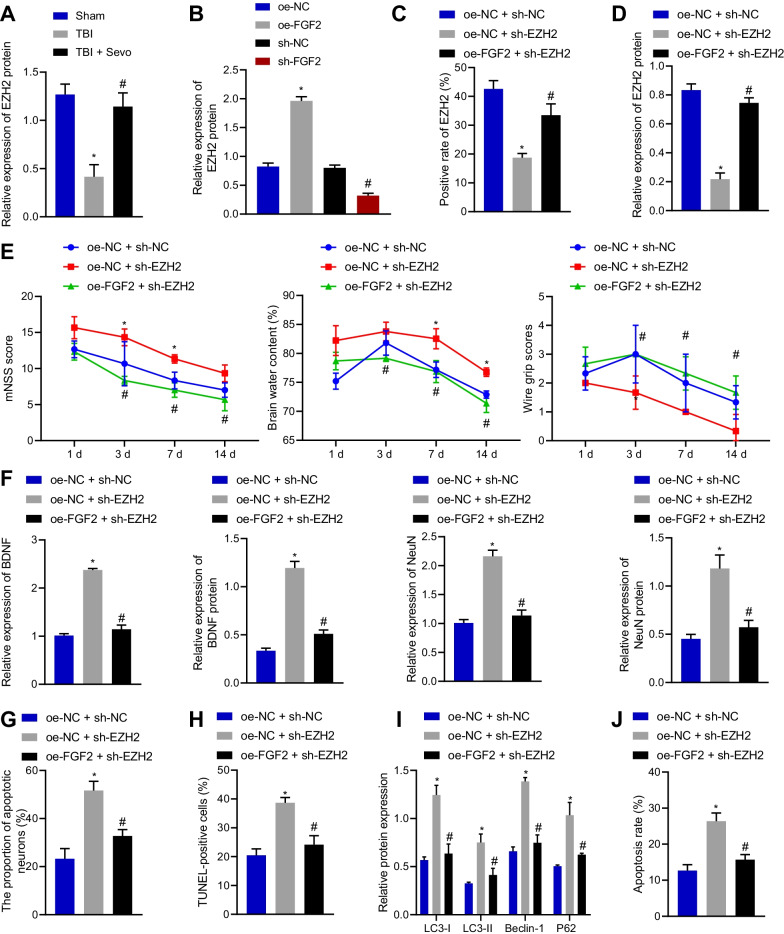
Sevo activates EZH2 expression via FGF2 to repress neurological deficits in TBI rats. **A** Western blot analysis of EZH2 expression in the cortical tissue of sham-operated, TBI, or Sevo-treated TBI rats. Sevo-treated TBI rats were treated with sh NC, sh FGF2, oe NC, or oe FGF2. **B** Western blot analysis of EZH2 expression in the cortical tissue of TBI rats. Sevo-treated TBI rats were treated with sh NC, sh EZH2, or sh EZH2 + oe FGF2. **C** Immunofluorescence analysis of EZH2 expression in the cortical tissue of TBI rats. **D** Western blot analysis of EZH2 expression in the cortical tissue of TBI rats. **E** Neurological function assessment by mNSS, brain water content evaluation and motor function score in TBI rats. **F** BDNF and NeuN expression measured by RT-qPCR and Western blot analysis in the cortical tissue of TBI rats. **G** Nissl staining of the neuronal damage in the cortical tissue of TBI rats. **H** TUNEL-positive cells in the cortical tissue of TBI rats. **I** Protein expression of autophagy-related genes (LC3-I, LC3-II, Beclin-1, and P62) in the cortical tissue of TBI rats detected by Western blot analysis. **J** Flow cytometric analysis of hippocampal neuronal apoptosis in the cortical tissue of TBI rats. *p < 0.05 vs. sham-operated rats or Sevo-treated TBI rats treated with oe NC or oe NC + sh NC; ^#^*p* < 0.05 vs. TBI rats or Sevo-treated TBI rats treated with sh NC or oe NC + sh EZH2. *n* = 12 for rats upon each treatment

The aforesaid results suggested that Sevo could increase FGF2 expression in TBI rats to prevent TBI. Next, we further investigated the mechanism by which FGF2 exerted neuroprotective functions during TBI. Western blot analysis data revealed a reduction in the EZH2 expression in the cortical tissue of TBI rats, while Sevo treatment elevated its expression (Fig. 3A; Additional file 2: Fig. S1G). In addition, overexpression of FGF2 promoted the EZH2 expression, which was negated following FGF2 silencing (Fig. 3B; Additional file 2: Fig. S1H), suggesting that FGF2 can positively regulate the expression of EZH2.

Based on the results of immunofluorescence staining (Fig. 3C) and Western blot analysis (Fig. 3D), sh EZH2 treatment contributed to a decline of EZH2 expression in the cortical tissue of Sevo-treated TBI rats, which was rescued by further oe FGF2 treatment.

Silencing EZH2 elevated mNSS and brain water content while reducing the motor function score in Sevo-treated TBI rats, which was neutralized by further overexpression of FGF2 (Fig. 3E). Furthermore, RT-qPCR and Western blot analysis results displayed that BDNF and NeuN expression was reduced upon silencing of EZH2 in the cortical tissue of Sevo-treated TBI rats, which was normalized by further overexpression of FGF2 (Fig. 3F). The results of Nissl and TUNEL staining documented that neuronal damage and apoptosis were augmented in the cortical tissue of Sevo-treated TBI rats following silencing of EZH2, which was annulled after further overexpression of FGF2 (Fig. 3G, H).

Western blot analysis exhibited upregulation of LC3-I, LC3-II, Beclin-1, and P62 in Sevo-treated TBI rats with silencing of EZH2, which was nullified by additional overexpression of FGF2 (Fig. 3I). Moreover, flow cytometric analysis described an enhancement in the neuronal apoptosis in the presence of EZH2 silencing, the effect of which was abolished by overexpression of FGF2 (Fig. 3J). In summary, Sevo upregulated FGF2 expression to activate EZH2, thereby arresting neurological deficits in TBI rats.

### Sevo inhibited HES1 expression by upregulating EZH2 and promoting HES1 promoter methylation

**Fig. 4:**
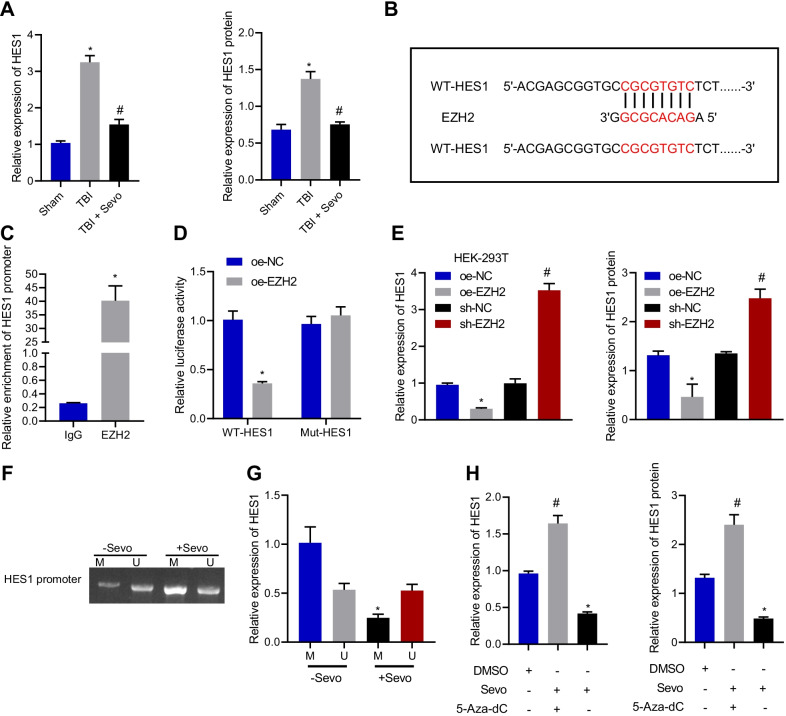
Sevo decreases HES1 expression by orchestrating HES1 promoter methylation via EZH2. **A** HES1 expression in the cortical tissue of sham-operated, TBI, or Sevo-treated TBI rats measured by RT-qPCR and Western blot analysis. **B** JASPAR database predicting binding sites of EZH2 to HES1 promoters. **C** Dual luciferase reporter assay validating the interaction between HES1 promoter and EZH2. **D** ChIP assay for EZH2 binding to HES1 promoter in the hippocampal neuron. **E** RT-qPCR and Western blot analysis of HES1 expression in hippocampal neurons after overexpression or silencing of EZH2. **F** HES1 promoter methylation in hippocampal neurons assessed by MSP. **G** Expression of HES1 in hippocampal neurons treated with Sevo or 5-Aza-dC determined by RT-qPCR. **H** Expression of HES1 in hippocampal neurons after treatment with Sevo or 5-Aza-dC detected by RT-qPCR and Western blot analysis. *p < 0.05 vs. sham-operated rats, oe NC-treated 293T cells, cell lysate incubated with IgG antibody, or hippocampal neurons treated with oe NC, Sevo, or DMSO. ^#^*p* < 0.05 vs. TBI rats or hippocampal neurons treated with sh NC. Cell experiments were conducted three times independently

EZH2 has been documented to upregulate HES1 in erythroid cells [13]. To investigate how EZH2 orchestrated HES1 expression, RT-qPCR and Western blot analysis were conducted, the results of which depicted that compared with sham-operated rats, HES1 expression was obviously elevated in the cortical tissue of TBI rats while it was diminished in the cortical tissue TBI rats after Sevo treatment (Fig. 4A). To determine whether EZH2 suppressed HES1 expression by binding to the promoter of HES1, we first predicted the WT or MUT binding sites between EZH2 and HES1 promoter through JASPAR database (Fig. 4B). Additionally, dual luciferase reporter assay data displayed that overexpression of EZH2 decreased the luciferase activity of WT-HES1 without altering that of MUT-HES1 (Fig. 4C). ChIP experiment results showed that EZH2 was highly enriched on the promoter region of HES1 (Fig. 4D), confirming EZH2 binding to the promoter of HES1. Furthermore, the results of RT-qPCR and Western blot analysis suggested that overexpression of EZH2 notably reduced HES1 expression, but silencing of EZH2 led to an opposite result (Fig. 4E). MSP experimental results documented that there was hyper-methylation in the promoter of HES1 under Sevo treatment (Fig. 4F). As illustrated by RT-qPCR, Sevo treatment caused downregulation of HES1 in hippocampal neurons (Fig. 4G). In addition, RT-qPCR and Western blot analysis results exhibited that the decreased HES1 expression by Sevo treatment was reversed by treatment with 5-aza-2′-deoxycytidine (5-aza-CdR; a methylation inhibitor) (Fig. 4H). To sum up, Sevo could upregulate EZH2 and promote HES1 promoter methylation, thus inhibiting the expression of HES1.

### Sevo depressed neurological injury induced by TBI in rats by downregulating HES1 via activation of FGF2/EZH2 axis

**Fig. 5:**
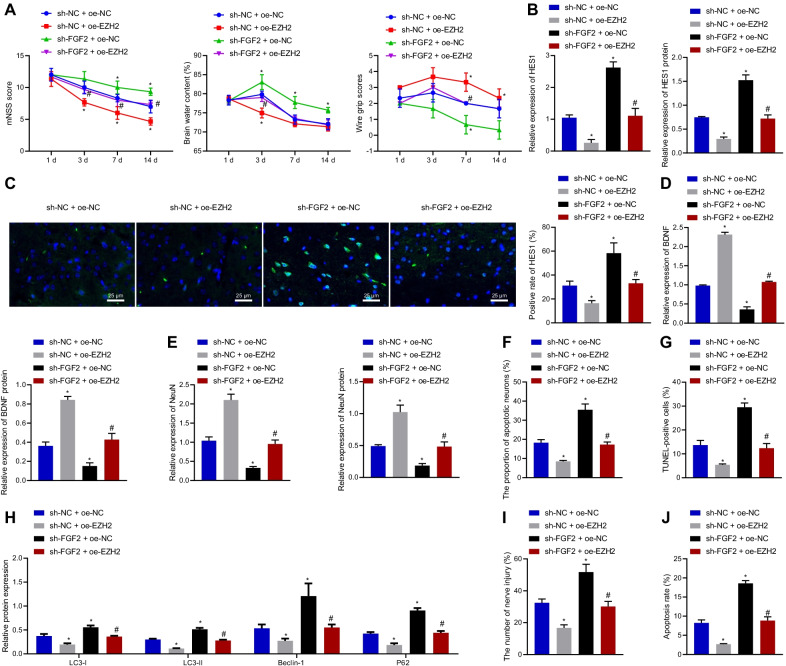
Sevo reduces HES1 expression by activating FGF2/EZH2 axis to suppress neurological injury in TBI rats. Sevo-treated TBI rats were treated with sh NC + oe NC, sh NC + oe EZH2, sh FGF2 + oe NC, or sh FGF2 + oe EZH2. **A** Neurological function assessment by mNSS, brain water content evaluation and motor function score in TBI rats. **B** RT-qPCR and Western blot analysis detection of HES1 expression in the cortical tissue of TBI rats. **C** Immunofluorescence staining analysis of HES1 expression in the cortical tissue of TBI rats (scale bar: 25 μm). **D** RT-qPCR and Western blot analysis detection of BDNF expression in the cortical tissue of TBI rats. **E** RT-qPCR and Western blot analysis of NeuN expression in the cortical tissue of TBI rats. **F** Neuronal damage assessed by Nissl staining. **G** TUNEL-positive cells in the cortical tissue of TBI rats. **H** Protein expression of autophagy-related genes (LC3-I, LC3-II, Beclin-1, and P62) in the cortical tissue of TBI rats detected by Western blot analysis. TBI hippocampal neurons were treated with sh NC + oe NC, sh NC + oe EZH2, sh FGF2 + oe NC, or sh FGF2 + oe EZH2. **I** Neuronal damage assessed by cell immunofluorescence staining. **J** The apoptosis of hippocampal neurons measured by flow cytometry. In panel **A**–**H**, *n* = 12 for rats upon each treatment. *p < 0.05 vs. Sevo-treated TBI rats or hippocampal neurons treated with sh NC + oe NC. ^#^*p* < 0.05 vs. Sevo-treated TBI rats or hippocampal neurons treated with sh NC + oe EZH2. Cell experiments were conducted three times independently

The abovementioned results have reported that Sevo could promote EZH2 expression via FGF2, thereby exerting neuroprotective functions during TBI by downregulating HES1. To further investigate the effects of Sevo on neurological injury in TBI rats by mediating FGF2/EZH2/HES1 axis, Sevo-treated TBI rats were randomly assigned into four groups via different lentivirus injection. As displayed in Fig. 5A, mNSS and brain water content were decreased while the motor function score was increased by treatment with sh NC + oe EZH2 in Sevo-treated TBI rats, which was abrogated by treatment with sh FGF2 + oe NC. Relative to treatment with sh NC + oe EZH2, treatment with sh FGF2 + oe EZH2 led to higher mNSS and brain water content and lower motor function score. The results of Western blot analysis and immunofluorescence staining documented reduction of HES1 expression after treatment with sh NC + oe EZH2 in the cortical tissue of Sevo-treated TBI rats, which was counteracted by sh FGF2 + oe NC. Additionally, sh FGF2 + oe EZH2 induced higher HES1 expression that sh NC + oe EZH2 (Fig. 5B, C). As manifested by RT-qPCR and Western blot analysis, treatment with sh NC + oe EZH2 led to an increase of BDNF and NeuN expression in the cortical tissue of Sevo-treated TBI rats, whereas treatment with sh FGF2 + oe NC restored these trends. However, lower BDNF and NeuN expression was noted in the presence of sh FGF2 + oe EZH2 than treatment with sh NC + oe EZH2 (Fig. 5D, E). The Nissl and TUNEL staining results (Fig. 5F, G) displayed that the neuronal damage and apoptosis were attenuated by sh NC + oe EZH2 treatment, which was neutralized by sh FGF2 + oe NC. In addition, combined treatment with sh FGF2 and oe EZH2 augmented the neuronal damage and apoptosis than oe EZH2 alone.

Moreover, the results of Western blot analysis exhibited a decline in the expression of LC3-I, LC3-II, Beclin-1, and P62 in the cortical tissue of Sevo-treated TBI rats following treatment with sh NC + oe EZH2, which was negated by FGF2 silencing. Combined treatment with sh FGF2 and oe EZH2 elevated the expression of LC3-I, LC3-II, Beclin-1, and P62 than oe EZH2 alone (Fig. 5H).

As described by the results of cell immunofluorescence staining (Fig. 5I), the hippocampal neuronal damage was diminished in Sevo-treated TBI rats treated with sh NC + oe EZH2, which was normalized after additional silencing of FGF2. Flow cytometric data (Fig. 5J) indicated that cell apoptosis was lowered by treatment with sh NC + oe EZH2, while the effect of EZH2 overexpression could be reversed by FGF2 silencing. In conclusion, Sevo activated the FGF2/EZH2 axis to decrease HES1 expression, thus inhibiting neurological injury in TBI rats. FGF2 acted not by increasing the protein level of EZH2, but by another mechanism.

## Discussion

TBI can contribute to the reduction of memory loss or forgetfulness, level of awareness or consciousness, other neurological or neuropsychological abnormalities, and even death, with annual increase of incidence rate of TBI [21]. TBI also is an epigenetic risk factor for neurological diseases, such as Alzheimer’s disease, Parkinson’s disease, and depression, which trigger higher demands for institutional and long-term care [22]. Additionally, the neuroprotective role of Sevo has been identified in ischemic brain injury [23]. In this context, our research aimed to explore whether Sevo coffered neuroprotection against TBI and related potential mechanisms. Consequently, our data elucidated that Sevo might elevate FGF2 expression to enhance the methylation modification ability of EZH2 binding to HES1 promoter, thus diminishing HES1 transcription and then alleviating neuronal apoptosis and brain edema in TBI rats.

The initial finding in our research was that Sevo reduced brain edema and neuronal apoptosis and autophagy and improved the neurological deficits in TBI rats. Consistently, a research conducted by He et al. manifested that Sevo postconditioning was capable of repressing neuronal apoptosis and brain edema and improved nerve function in TBI rats, while the neuroprotective effects of Sevo postconditioning were reversed by the 3-MA treatment [8]. Sevoflurane postconditioning has been shown to protect the heart from ischemia–reperfusion (I/R) injury by restoring intact autophagic flux [24]. Meanwhile, a recent study has demonstrated that Sevo postconditioning can attenuate brain damage by inhibiting neuronal autophagy and apoptosis in cerebral I/R rats [25]. In addition, there exist mounting researches elaborating the neuroprotection of Sevo in numerous brain injuries. For instance, a prior work uncovered that Sevo exerted neuroprotective effects on electromagnetic pulse-induced brain injury by decreasing neuronal apoptosis and attenuating neurological deficits in rats [26]. Also, Sevo was able to improve the neurological scores, motor coordination, and neuronal injury to induce neuroprotection against cerebral ischemic brain injury [27]. In line with our results, another research elucidated that Sevo contributed to reduction in neuronal apoptosis and improvement in long-term cognitive function in neonatal rats after hypoxic-ischemic brain injury [28]. Therefore, these findings confirmed the neuroprotection of Sevo in TBI.

McNiel et al. [11] analyzed the cancer genome map (TCGA) and Oncomine. The data showed that KDM2B was related to EZH2 expression. Their further studies showed that FGF-2 could activate DYRK1A, phosphorylate CREB, and induce expression of histone H3K36me2/ME1 demethylase KDM2B. Kottakis et al. [29] reported that KDM2B can cooperate with EZH2 to regulate cell proliferation, migration, angiogenesis, transformation, maintenance, and self-renewal of stem cells and human progenitor cells. Its cellular biological role depends on it as a group of main regulators. It can target miRNAs of several components of polyfilaments, including miR-101, activate a switch and stably upregulate EZH2 [30]. Through a series of animal and cell experiments, we further observed that Sevo triggered FGF2 upregulation, and that FGF2 overexpression diminished brain edema, neurological deficits, and neuronal apoptosis and autophagy in TBI rats. Coincidently, a previous work manifested the alleviation of blood–brain barrier damage and brain edema in rats with TBI after overexpressing FGF2 [31]. Similarly, another work also clarified that FGF2 overexpression resulted in attenuation of brain edema and neurological deficits and enhancement of the number of surviving neurons in injured cortex and the ipsilateral hippocampus of TBI rats, as well as lowered neuronal apoptosis and autophagy [17]. In addition, FGF2 overexpression reduced excessive neuronal autophagy and apoptosis to offer neuroprotection against transient global cerebral ischemia in rats [32].

More importantly, it has been detected in a prior study that FGF2 is capable of upregulating EZH2 in bladder cancer cells [29], which was partially consistent with our result. Specially, our result discovered that EZH2 could bind to the promoter of HES1 and decreased HES1 expression through its methylase function. Concordantly, a prior work indicated that EZH2 overexpression could contribute to inhibition of HES1 expression in erythroid cells [13]. Further analysis in our research illustrated that EZH2 overexpression depressed brain edema, neurological deficits, and neuronal apoptosis and autophagy in TBI rats by downregulating HES1. It was noted in a previous research that Sevo could obviously augment EZH2 expression to reduce over-activated autophagy, thus having neuroprotective effects in neonatal rats with hypoxic-ischemic cerebral injury [12]. Besides, the HES1 upregulation has been detected in mice with TB1 by the research of Wang et al. [33]. Consistently, a prior research suggested that HES1 overexpression was involved in promotion of neuronal apoptosis in rats with spinal cord injury [34].

## Conclusions

**Fig. 6:**
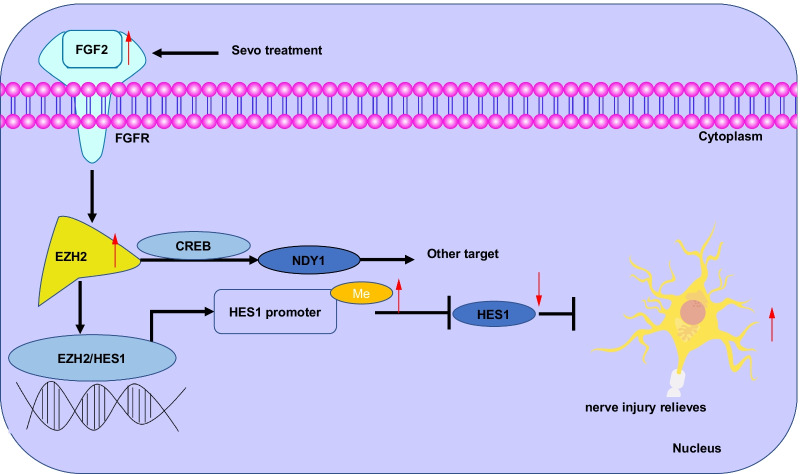
Schematic diagram of the mechanism by which Sevo affects TBI via the FGF2/EZH2/HES1 axis. Sevo upregulates FGF2 to elevate EZH2 expression, promote the methylation of the promoter of HES1, and inhibit the transcription of HES1, thereby inhibiting neuronal apoptosis and autophagy, and ultimately promoting the repair of neurological function in TBI rats

Taken together, the findings from the present study suggest that Sevo attenuated neuronal apoptosis and autophagy to confer neuroprotection against TBI in rats by downregulating HES1 via activation of the FGF2/EZH2 axis (Fig. 6). These findings may provide a better understanding regarding the mechanism of Sevo and FGF2 in TBI. Moreover, prospective studies that could translate these findings regarding the role of Sevo-upregulated FGF2 in TBI into clinical applications will be greatly beneficial.

## Supplementary Information

**Additional file 1: Table 1.** Rat grouping. **Table 2.** Information of antibodies. **Table 3.** Primer sequences for RT-qPCR. **Table 4.** Primer sequences for ChIP assay. **Table 5.** Primer sequences for MSP assay.

**Additional file 2: Fig S1.** Original Western blots. **A** The protein expression of BDNF detected by Western blot analysis in the cortical tissue of rats. **B** Western blot analysis of the protein expression of NeuN in the cortical tissue of rats. **C** Western blot analysis of FGF2 expression in the cortical tissue of sham-operated, TBI, or Sevo-treated TBI rats. Sevo-treated TBI rats were treated with sh NC, sh FGF2, oe NC, or oe FGF2. **D** Western blot analysis of FGF2 expression in the cortical tissue of TBI rats. **E** The expression of BDNF determined by Western blot analysis in the cortical tissue of TBI rats. **F** Western blot analysis of the expression of NeuN in the cortical tissue of TBI rats. **G** Western blot analysis of EZH2 expression in the cortical tissue of sham-operated, TBI, or Sevo-treated TBI rats. Sevo-treated TBI rats were treated with sh NC, sh FGF2, oe NC, or oe FGF2. H, Western blot analysis of EZH2 expression in the cortical tissue of TBI rats.

## Data Availability

The original contributions presented in the study are included in the article/supplementary material, further inquiries can be directed to the corresponding author.

## Abbreviations

TBI: Traumatic brain injury
Sevo: Sevoflurane
FGF2: Fibroblast growth factor 2
EZH2: Enhancer of zeste homolog 2
HES1: Hairy Enhancer of Split 1
mNSS: Modified Neurological Severity Score
NeuN: Neuronal nuclear
GAPDH: Glyceraldehyde-3-phosphate dehydrogenase
IgG: Immunoglobulin G
DAPI: 4′,6-Diamidino-2-phenylindole
