# Supplementary material for: The neuroprotective mechanism of sevoflurane in rats with traumatic brain injury via FGF2
Source: J Neuroinflammation. 2022 Feb 17;19:51. doi: 10.1186/s12974-021-02348-z (PMC8855620; doi:10.1186/s12974-021-02348-z)
Supplement: Supplementary file 1 — Additional file 1: Table 1. Rat grouping. Table 2. Information of antibodies. Table 3. Primer sequences for RT-qPCR. Table 4. Primer sequences for ChIP assay. Table 5. Primer sequences for MSP assay. [file 12974_2021_2348_MOESM1_ESM.docx]

**Additional Table 1** Rat grouping

| Groups | Treatment |
| --- | --- |
| Sham group | Sham-operation |
| TBI group | TBI modeling using a modified Feeney's free-falling epidural percussion method |
| TBI + Sevo group | 1-h inhalation of Sevo after TBI modeling |
| oe FGF2 group | Intracerebral injection of oe FGF2 two days before TBI modeling and 1-h inhalation of Sevo after TBI modeling |
| oe NC group | Intracerebral injection of oe NC two days before TBI modeling and 1-h inhalation of Sevo after TBI modeling |
| sh NC group | Intracerebral injection of sh NC two days before TBI modeling and 1-h inhalation of Sevo after TBI modeling |
| sh FGF2 group | Intracerebral injection of sh FGF2 two days before TBI modeling and 1-h inhalation of Sevo after TBI modeling |
| sh EZH2 group | Intracerebral injection of sh EZH2 two days before TBI modeling and 1-h inhalation of Sevo after TBI modeling |
| sh NC + oe NC group | Intracerebral injection of sh NC + oe NC two days before TBI modeling and 1-h inhalation of Sevo after TBI modeling |
| sh FGF2 + oe NC group | Intracerebral injection of sh FGF2 + oe NC two days before TBI modeling and 1-h inhalation of Sevo after TBI modeling |
| sh FGF2 + oe EZH2 group | Intracerebral injection of sh FGF2 + oe EZH2 two days before TBI modeling and 1-h inhalation of Sevo after TBI modeling |
| oe FGF2 + sh EZH2 group | Intracerebral injection of oe FGF2 + sh EZH2 two days before TBI modeling and 1-h inhalation of Sevo after TBI modeling |
| sh NC + oe EZH2 group | Intracerebral injection of sh NC + oe EZH2 two days before TBI modeling and 1-h inhalation of Sevo after TBI modeling |

Notes: TBI, traumatic brain injury; Sevo, sevoflurane; oe, overexpression; NC, negative control; sh, short hairpin RNA; FGF2, fibroblast growth factor 2; EZH2, enhancer of zeste homolog 2.

**Additional Table 2** Information of antibodies

| Protein | Product codes and Company |
| --- | --- |
| NeuN | ab177487, 1:1000, Abcam, Cambridge, UK |
| MAP2 | ab183830, 1:1000, Abcam, Cambridge, UK |
| FGF2 | ab208687, 1:1000, Abcam, Cambridge, UK |
| EZH2 | ab186006, 1:1000, Abcam, Cambridge, UK |
| IgG | ab172730, 1:1000, Abcam, Cambridge, UK |
| HES1 | ab108937, 1:1000, Abcam, Cambridge, UK |
| LC3 | ab192890, 1:1000, Abcam, Cambridge, UK |
| LC3-I | ab232940, 1:1000, Abcam, Cambridge, UK |
| LC3-II | ab2394161, 1:1000, Abcam, Cambridge, UK |
| P62 | ab240635, 1:1000, Abcam, Cambridge, UK |
| GAPDH | ab9485, 1:1000, Abcam, Cambridge, UK |
| BDNF | ab108319, 1:1000, Abcam, Cambridge, UK |

Notes: NeuN, Neuronal nuclear antigen; MAP2, Microtubule-associated Protein 2; FGF2, fibroblast growth factor 2; EZH2, enhancer of zeste homolog 2; IgG, Immunoglobulin G; HES1, Hairy Enhancer of Split 1; LC3, Light Chain 3; GAPDH, glyceraldehyde-3-phosphate dehydrogenase; BDNF, Brain-derived neurotrophic factor.

**Additional Table 3** Primer sequences for RT-qPCR

| Targets | Sequences (5’-3’) |
| --- | --- |
| HES1 | F: TGAAGGATTCCAAAAATAAAATTCTCTGGG |
|  | R: CTCTTCTCCATGATAGGCTTTGATGAC |
| FGF2 | F: ACCCGGCCACTTCAAGG |
|  | R: GATGCGCAGGAAGAAGCC |
| EZH2 | F: TCTCACCAGCTGCAAAGTGT |
|  | R: AGAATCAGTTGGTGATGTTCTGT |
| GAPDH | F: CTGGCCAAGGTCATCCATGAC |
|  | R: CTTGCCCACAGCCTTGGCAG |

Notes: FGF2, fibroblast growth factor 2; EZH2, enhancer of zeste homolog 2; HES1, Hairy Enhancer of Split 1; GAPDH, glyceraldehyde-3-phosphate dehydrogenase; R, reverse; F, forward; RT-qPCR, reverse transcription quantitative polymerase chain reaction.

**Additional Table 4** Primer sequences for ChIP assay

| Names | Sequences (5’-3’) |
| --- | --- |
| HES1 (pro) | F: CTCTTCCTCCCATTGGCTGA |
|  | R: GCACCAGCTCCAGATCCTGT |
| Thp (pro) | F: GGTGGATGGTGTGGTCACAAC |
|  | R: GGTCTTGACACACCAGCTTT |

Notes: HES1, Hairy Enhancer of Split 1; pro, promoter; R, reverse; F, forward; ChIP, Chromatin immunoprecipitation.

**Additional Table 5** Primer sequences for MSP assay

| Names | Sequences (5’-3’) |
| --- | --- |
| Methylated | F: CGGAGGTAGGAGGTTGATTC |
|  | R: TTAAAATTTTCACTCGACCG |
| Unmethylated | F: GAGTGGAGGTAGGAGGTTGATTT |
|  | R: TTAAAATTTTCACTCAACCAAAA |
